# Influence of Identity Development on Weight Gain in Adolescent Anorexia Nervosa

**DOI:** 10.3389/fpsyt.2022.887588

**Published:** 2022-05-26

**Authors:** Lynn I. Budde, Simon Wilms, Manuel Föcker, Anke Dalhoff, Joerg M. Müller, Ida Wessing

**Affiliations:** Department of Child and Adolescent Psychiatry, University Hospital Münster, Münster, Germany

**Keywords:** anorexia nervosa, adolescence, identity, personality, inpatient treatment, weight gain

## Abstract

**Background:**

Anorexia Nervosa (AN) typically begins during early adolescence, an important phase of personality development. A substantial proportion of adolescent AN patients shows impaired personality functioning, which might be a relevant but understudied aspect of illness severity. The developmental status of identity as key element of personality is suggested to influence inpatient treatment outcome in adolescents with AN.

**Methods:**

This study analyzed existing data of *N* = 60 adolescents with AN. Multilevel models assessed the influence of identity functioning, measured by the *Assessment of Identity Development in Adolescence* (AIDA) at admission, on weight gain [BMI (body mass index), BMI-SDS (BMI standard deviation score)] during 10 weeks of inpatient treatment. Moreover, the influence of other indicators of illness severity, i.e., eating disorders and comorbid psychopathologies, was explored.

**Results:**

As expected, higher AIDA scores negatively influenced the course of weight gain. A similar effect was observed for other psychopathology measures, especially body image distortion. In general, higher weight at admission was associated with less weight gain. Higher weight at admission was also predicted by higher other psychopathology measures, but not AIDA scores.

**Conclusion:**

The course of weight gain during inpatient treatment was hampered in adolescent AN patients who have difficulties developing a stable identity. Unlike other aspects of psychopathology, this was independent of the initial weight. Thus, in addition to the level of underweight and other aspects of psychopathology, difficulties in identity development constitute a relevant aspect of illness severity in AN. This recommends consideration of identity development during treatment.

## Introduction

Anorexia nervosa (AN) is characterized by significantly low body weight, an intense fear of gaining weight and/or behavior that interferes with weight gain (1) and is associated with a high risk of morbidity and mortality (2, 3). The lifetime prevalence of AN is about 0.3–0.6% and it predominantly affects female adolescents and young women (1, 4). One risk factor for the onset of AN during coming of age might be difficulties in the accomplishment of age-related tasks, like the development of autonomy and a stable identity (5, 6).

The creation of a stable identity is a major task during adolescence (7–9). Erikson defines identity as the central organization principle of the constantly changing personality (9). Identity enables continuity of the self (“self-sameness”) and differentiation from others (“uniqueness”) and is a key element in psychosocial integration. During adolescence individuals are confronted with profound physical and psychological changes and difficulties with self identification, as one's and others' perception of the self often differ. This can result in a temporary identity crisis, whereas the continuity of the self remains stable over time (10, 11). Overcoming identity crises during adolescence and young adulthood enables self-reflection, independence, and healthy social interactions (11). Beyond a temporary crisis, individuals may develop a pathological and more severe identity diffusion. It creates a feeling of chronic emptiness, inconsistency, and other signs of weakness of the self (12, 13). Identity diffusion is seen as a basis for all severe personality disorders (PD) (10, 11, 14, 15). This is also reflected in the current fifth edition of the DSM (DSM-5) (1) where identity is a key element of personality functioning (see the alternative model for PD section 3) (1).

In adult AN patients, PDs are among the most frequent comorbid conditions and a recent meta-analysis revealed its occurrence in 49% of AN cases. The most common specific PD diagnoses were Cluster C and Borderline PDs (16). Importantly, comorbid PDs in AN are associated with worse outcomes, e.g., elevated mortality (17). Comorbid Borderline PD in AN was associated with fewer treatment-related improvements of global functioning and body dissatisfaction (18). Moreover, comorbid PDs were associated with more frequent treatment drop out and less weight gain specifically in young adult AN patients aged 17–24 (19). Less is known about PDs in adolescent AN patients, probably because the DSM-IV had restricted PD diagnosis to adults (20). Although the DSM-5 now enables PD diagnoses in individuals under the age of 18 (1), it is still controversial whether it is adequate to diagnose a PD in adolescence (21) or specifically in adolescent AN patients (22, 23). Some authors claim adolescence is *per se* a turbulent and uncertain time which complicates the differentiation between healthy and ill (12). Morover, many clinicians are hesitant to diagnose PDs in adolescence as it might be labeling and stigmatizing and could negatively affect the patients' development (12). However, several studies suggest that PD symptoms are apparent and sufficiently stable during adolescence (24, 25). Indeed, two studies using (semi-)structured clinical interviews in adolescent AN patients revealed comorbid PDs in 22–28% of cases (26, 27) and a longitudinal study found PDs in 25% of adolescent AN cases at a 10 year follow-up (28). Thus, PD symptoms occur in a relevant proportion of adolescent AN patients. In light of the above uncertainties regarding the validity and stability of PDs during this vulnerable phase of personality development (29), we suggest that the DSM-5 dimensional model for PD might be particularly useful in adolescent AN patients. According to this model, identity is a central element of personality. There is first evidence, that the prescence of PD symptoms is related to illness severity also in this younger group (26). Conversely, impairment of identity development is also seen as a core vulnerability for the development and persistence of AN (29–31). While the direction of a possible causal relationship between identity impairment and AN remains open, these reports substantiate the possible importance of identity development for the severity of AN.

A substantial proportion of severely ill adolescent AN patients needs intensive care, including inpatient treatment, with the primary goal being weight restoration (32). Indeed, weight gain within the first weeks is a key outcome variable in AN inpatient treatment (33, 34). However, not all patients achieve clinically significant weight changes (35) and individual courses of weight gain vary substantially (36). Thus, it is of great interest to determine relevant clinical parameters that influence the course of weight gain and might call for special attention during treatment.

Until now, among the most studied clinical parameters known to influence inpatient treatment outcome in AN are several commonly used indices of illness severity, including parameters such as weight or body mass index (BMI) at admission, duration of illness, severity of eating disorder (ED) symptoms, body image distortion (BID) and the presence of comorbid anxiety and depression [adolescents: (34, 35, 37, 38); adults: (39)]. Based on the above data on PD in AN, we suggest that the level of personality function, and specifically identity development, can be used as an additional important index of illness severity. Still, it has been comparatively less studied, particularly in adolescent AN patients (40).

The aim of this study was thus to examine the influence of identity development on weight gain during inpatient treatment in adolescents with AN. To this end, identity development was measured with the self-report questionnaire *Assessment of Identity Development in Adolescence* (AIDA) (9) at the beginning of the treatment and multilevel models predicted its influence on changes of BMI and BMI standard deviation score (BMI-SDS) over 10 weeks. We hypothesized that the increase of weight over time would be weakened by lower levels of identity development, i.e., higher AIDA scores. Moreover, if the level of identity development is indeed a core vulnerability of the illness (30), it might be a specifically strong predictor of weight gain. Thus, for comparison purposes, we additionally explored the predictive value of several other indices of illness severity, in particular ED symptoms, BID, and comorbid anxiety and depression.

## Materials and Methods

### Participants and Treatment Concept

We retrospectively examined all individuals with AN who were consecutively admitted to inpatient treatment in the Department of Child and Adolescent Psychiatry, University Hospital Münster, Germany over the time period of January 2017 until March 2021. The study was approved by the ethics committee of the Medical Association of Westphalia-Lippe and the University of Münster. Inclusion criteria were female sex and a primary diagnosis of AN, comprising restrictive type, bulimic type or atypical type. Patients were first selected based on diagnoses according the ICD-10-GM (41), as this is mandatory in the German health system. Based on the patients' records, an independent clinical expert retrospectively confirmed the AN criteria of the DSM-5 (1). The individuals had to have a hospitalization period of at least 10 weeks. This duration was chosen to optimize both the period under review and the number of included patients. If patients had multiple hospital stays during the studied time period, we only included data of the first stay. Patients with comorbid neurodevelopmental, schizophrenia spectrum, or bipolar disorders were excluded. Moreover, we excluded one individual diagnosed with Turner syndrome and one individual with gender dysphoria to avoid possible effects of the hormone therapy. Individuals were treated according to a disorder-specific concept including a phased multimodal treatment plan. Patients received individual, family, and group therapy, body psychotherapy, art therapy, and participated in group activities such as cooking, eating out and guided exercises. Moreover, the treatment concept included an agreed target weight at the 25th age-percentile and a weekly weight gain of 500 g. Depending on the last outpatient calorie intake, calories were increased from at least 800 kcal to more than 2,000 kcal in the first few days.

### Procedure

The analyzed data were collected retrospectively and anonymously from the internal patient system. All data were initially collected as part of the clinical routine. Individuals with AN completed a set of standard diagnostics consisting of clinical questionnaires and a behavioral test of body image distortion (see Table 1 and Section Questionnaires and Body Size Estimation Task below). These measures were collected at the beginning of treatment. The mean time between admission and completion of the *Assessment of Identity Development in Adolescence* (AIDA) was *M* = 15.00 days (*SD* = 27.40). Body weight and height were measured according to a standard operating procedure (SOP) using the same calibrated scale and stadiometer for all study participants. All adolescents were weighed in underwear without shoes. Weight was measured at least once a week and height once a month. The survey period ended after 10 weeks. Some patients were discharged at that time and others continued inpatient treatment. All sociodemographic and clinical information, such as age, onset, duration of inpatient treatment, comorbidities, and medication were available from the patient records. Using this data, BMI, BMI-SDS, and BMI percentiles were assessed. The calculations of age-adjusted BMI-SDS (*z*-scores) and BMI percentiles were based on the German Health Interview and Examination Survey for Children and Adolescents (KiGGS) data (42) for individuals aged under 18 and were based on the Kronmeyer Hauschild data (43) for individuals aged 18 or older.

**Table 1:** Descriptive statistics—questionnaires and behavioral test.

**Variable**	***M***	***M*** **95% CI**		***SD***
		***LL***	***UL***	
Age admission (years)	14.88	14.41	15.35	1,82
Height admission (cm)	166.77	164.70	168.84	8.01
Weight admission (kg)	41.40	39.96	42.84	5.58
BMI admission	14.84	14.50	15.18	1.32
BMI week 10	16.70	16.35	17.05	1.37
AIDA diffusion (total score)	96.00	87.69	104.31	32.17
AIDA discontinuity	43.95	39.96	47.94	15.44
AIDA incoherence	52.05	47.20	56.90	18.76
EDI-C	215.45	200.92	229.98	56.26
BSQ	112.45	101.49	123.41	42.43
BDI-II	24.48	21.71	27.26	10.74
SCARED	29.52	25.99	33.04	13.64
BID-CA (total score)	131.46	126.64	136.28	16.79
BID-CA arm	124.93	119.76	130.09	17.98
BID-CA waist	137.50	131.48	143.51	20.93
BID-CA thigh	131.96	125.71	138.21	21.75

*M and SD represent mean and standard deviation, respectively. LL and UL indicate the lower and upper limits of the 95% confidence interval for the mean, respectively. BMI, Body mass index; AIDA, Assessment of Identity Development in Adolescence; EDI-C, Eating Disorder Inventory for Children; BSQ, Body Shape Questionnaire; BDI-II, Beck-Depression-Inventory-II; SCARED, Screen for Child Anxiety Related Emotional Disorders; BID-CA, Test for Body Image Distortion in Children and Adolescents*.

### Questionnaires and Body Size Estimation Task

#### Assessment of Identity Development in Adolescence (AIDA)

The AIDA (9) is a German questionnaire assessing identity integration (i.e., healthy identity development) and identity disturbance (i.e., identity crisis, and identity diffusion) in adolescence (12, 19). All participants answered 58 items on a 5-point-scale (0 = *no*, 4 = *yes*). The AIDA consists of the total scale “diffusion” which is the sum of the two main scales “discontinuity” and “incoherence”. “Continuity” describes the emotional-intuitive “I”, meaning “self-sameness”, stability in relationships to others and oneself (including e.g., positive relationship to one's own body), and the ability to emotional self-reflection (e.g., “*I feel like I don't really belong anywhere*”; “*I often don't know how I feel right now*”). “Coherence” is defined as a cognitive-definitory “ME”, meaning a consistent self-image, autonomy, ego-strength, and the ability for cognitive self-reflection (e.g., “*I feel like I have different faces that do not fit together very well*”; “*I am confused about what kind of person I really am*”). Subscales divide into the classical psychological functions intrapersonal, interpersonal and mental representation (9). Reliability in this study was excellent for the total scale (α = 0.94) and good for the main scales (“discontinuity” α = 0.88 and “incoherence” α = 0.89). The multilevel model used the total scale “diffusion”.

#### Eating Disorder Inventory for Children (EDI-C)

The EDI-C (44) [German version: (45)] measures a wide range of symptoms and personality traits associated with EDs. The self-report questionnaire includes 91 items that are answered on a 6-point-scale (1 = *never*, 6 = *always*) (45). Reliability of the main scale was α = 0.96.

#### Body Shape Questionnaire (BSQ)

The BSQ (46) [German version: (47)] is a self-report questionnaire to measure body image distortion and body dissatisfaction. Patients answered 34 items on a 6-point-scale (1 = *never*, 6 = *always*) (47). The reliability was α = 0.97.

#### Beck-Depression-Inventory (BDI-II)

The BDI-II (48) [German version: (49)] assesses the severity of depressive symptoms. It comprises 21 items, each with four item-specific response options. The items are scored from 0 to 3, with a total score of 0 to 65 (0 = *no or minimal depression*, 29–65 = *severe depression*) (49). The reliability of the total score was α = 0.89.

#### Screen for Child Anxiety Related Emotional Disorders (SCARED)

The SCARED (50) [German version: (51)] is a screening for childhood anxiety disorders. It comprises 41 items on a 3-point scale (0 = *not true or hardly ever true*, 2 = *true or often true*) (51). The reliability was α = 0.93.

#### Body Image Distortion in Children and Adolescents (BID-CA)

The BID-CA (52) is a behavioral test that measures body size over- or underestimation. Patients estimate the circumference of their upper arm, waist, and thigh by forming a circle with a rope. The actual circumferences of the body parts are measured and compared to the patients' assessment. BID-CA indices for the different body parts and the mean value are calculated $\text{BID I arm}/\text{waist}/\text{thigh }=\;\frac{\text{perceived circumference }\left(\text{cm}\right)}{\text{actual circumference }\left(\text{cm}\right)}\times 100$. Prior studies, including our own study involving a partly overlapping sample (53), show that individuals with AN overestimate their body size (53). Test-retest reliability regarding a 1 week follow-up was determined by intraclass correlation (ICC) coefficients in a sample of *N* = 18 AN patients as follows: ICC_arm_ = 0.81; ICC_thigh_ = 0.74; ICC_waist_ = 0.62 (52).

### Statistical Analyses

All statistical analyses were performed with R (54) using the packages *ggplot2* (55) for data visualization, *lme4* (56) and *lmerTest* (57) for multilevel modeling.

The following analyses were conducted twice, once with the BMI and once with the BMI-SDS as dependent variable. The reason for that was that both variables offer specific benefits. The BMI represents weight changes more directly (a weight change of 1 kg is similarly weighted for each individual) and differentiates more accurately between individuals, whereas the BMI-SDS takes age into account, which is recommended for adolescents. However, the BMI-SDS has a greater statistical uncertainty, particularly regarding extreme values, which are frequent in individuals with AN (1, 42).

We hypothesized that higher AIDA scores would predict less increase of BMI/BMI-SDS over time. For each dependent variable (BMI, BMI-SDS), we computed separate models. First, we computed the intraclass coefficient (ICC). Second, we used a likelihood-ratio test to compare the random-intercept-fixed-slope-model with the AIDA score as level-2 predictor with the random-intercept-random-slope model. The model with random slopes should exhibit a significantly better fit to the data because we expected the individuals to vary regarding their BMI/BMI-SDS gain over time. Finally, for our hypothesis test the random-intercept-random-slope model was of particular interest: Since the AIDA score was *z*-standardized before analysis, the intercept c_00_ describes the predicted BMI/BMI-SDS value at the start of the survey period (time point of the first senior physician round 0–6 days after admission, for convenience only subsequently referred to as “admission”) given an average AIDA score. The standard deviation ŝ_0_ describes the variation of person-specific intercepts around the average intercept c_00_. The slope of time c_10_ describes the change of the predicted BMI/BMI-SDS values if time is increased by 1 week controlled for an average AIDA score. Since we hypothesized that patients would gain weight during their inpatient stay, we tested one-sidedly. Again, ŝ_1_ describes the variation of person-specific slopes around the average slope c_10_. Effects of the AIDA on person-specific intercepts and slopes are called c_01_ and c_11_, respectively. The c_01_ weight is the change in the predicted person-specific intercept (BMI/BMI-SDS at admission) if the AIDA score is increased by one standard deviation. The c_11_ cross-level interaction is the predicted change in person-specific slopes if the AIDA score is increased by one standard deviation. To confirm our hypothesis that higher AIDA scores would be associated with less BMI/BMI-SDS gain over time, this effect should be significantly negative. Again, we tested one-sidedly. Lastly, the correlation between person-specific intercepts and slopes provides information about the coherence between BMI/BMI-SDS at admission and its increase over time. All effects were estimated using a restricted maximum likelihood approach (REML). For both multilevel models we checked whether residuals were normally distributed using qq-plots. Furthermore, we visually inspected homoscedasticity by plotting fitted values against residuals.

Secondly, we explored if identity development measured with the AIDA questionnaire (total scale “diffusion”) is a specific predictor of the increase of BMI/BMI-SDS over time or if other indices of illness severity (other psychopathology parameters) have similar strong effects. To examine this, we repeated our above analyses with other self-report questionnaires and a behavioral measure of body image distortion instead of the AIDA. In detail these were EDI-C, BSQ, BDI-II, SCARED, and BID-CA indices (descriptive statistics see Table 1). As the other psychopathology parameters were not the primary focus of this paper, we refrained from formulating specific hypotheses and significance was tested two-sidedly.

## Results

### Participants

In total *N* = 14 individuals were excluded. Of these, 11 patients stayed >10 weeks, and one patient was male. The final sample consisted of *N* = 60 female adolescents with AN (restrictive type: *N* = 41, bulimic type: *N* = 13, atypical type/eating disorder not otherwise specified: *N* = 6). The sample included patients aged 9–18 years (*M* = 15.35, *SD* = 1.78). Mean onset of the disease was at age 14.28 (*SD* = 2.03). Average duration between disease onset and inpatient admission was 1.11 years (*SD* = 1.20). More than a half of the participants suffered from psychiatric comorbidities (*n* = 35; 58.33%). The most common were major depressive disorder (*n* = 24), anxiety disorder (*n* = 9), obsessive-compulsive disorder (*n* = 3), and trauma and stressor related disorders (*n* = 2; see Supplementary Table 1). About one third of the patients received psychiatric medication throughout their hospitalization (*n* = 21; 35.00%).

Descriptive statistics for the questionnaires and the behavioral test are presented in Table 1. Further information on the clinical relevance of the scores is provided in the Supplementary Data 1. Correlations are presented in Table 2.

**Table 2:** Correlations with confidence intervals.

**Variable**	**1**	**2**	**3**	**4**	**5**	**6**	**7**	**8**	**9**	**10**
1. BMI admission										
2. BMI-SDS admission	0.78**									
	[0.65, 0.86]									
3. BMI week 10	0.77**	0.55**								
	[0.64, 0.86]	[0.35, 0.71]								
4. BMI-SDS week 10	0.62**	0.83**	0.77**							
	[0.43, 0.75]	[0.74, 0.90]	[0.64, 0.86]							
5. Age *M* admission	0.07	−0.55**	0.14	−0.50**						
	[−0.18, 0.32]	[−0.70, −0.34]	[−0.12, 0.39]	[−0.67, −0.28]						
6. AIDA diffusion	0.18	0.05	0.02	−0.06	0.13					
	[−0.08, 0.42]	[−0.20, 0.30]	[−0.23, 0.27]	[−0.31, 0.20]	[−0.12, 0.38]					
7. EDI-C	0.27*	0.12	0.11	0.01	0.14	0.74**				
	[0.01, 0.49]	[−0.14, 0.36]	[−0.15, 0.35]	[−0.24, 0.26]	[−0.12, 0.38]	[0.59, 0.83]				
8. BSQ	0.48**	0.32*	0.28*	0.18	0.11	0.58**	0.84**			
	[0.26, 0.65]	[0.08, 0.53]	[0.02, 0.50]	[−0.08, 0.41]	[−0.14, 0.36]	[0.38, 0.73]	[0.75, 0.90]			
9. BDI-II	0.36**	0.21	0.22	0.12	0.11	0.74**	0.75**	0.79**		
	[0.12, 0.56]	[−0.04, 0.44]	[−0.04, 0.45]	[−0.14, 0.36]	[−0.14, 0.36]	[0.59, 0.83]	[0.61, 0.84]	[0.67, 0.87]		
10. SCARED	0.09	0.06	−0.07	−0.08	0.05	0.65**	0.57**	0.40**	0.54**	
	[−0.17, 0.34]	[−0.20, 0.31]	[−0.32, 0.19]	[−0.33, 0.18]	[−0.21, 0.30]	[0.47, 0.77]	[0.37, 0.72]	[0.17, 0.60]	[0.33, 0.69]	
11. BID-CA	0.28*	0.28*	0.09	0.13	−0.09	0.20	0.40**	0.51**	0.38**	0.18
	[0.01, 0.50]	[0.01, 0.50]	[−0.18, 0.34]	[−0.14, 0.38]	[−0.35, 0.18]	[−0.07, 0.44]	[0.15, 0.60]	[0.28, 0.68]	[0.12, 0.58]	[−0.09, 0.43]

M and SD are used to represent mean and standard deviation, respectively. Values in square brackets indicate the 95% confidence interval for each correlation. ^*^p < 0.05, ^**^p < 0.001. BMI, Body Mass Index; BMI-SDS, BMI standard deviation score; AIDA, Assessment of Identity Development in Adolescence; EDI-C, Eating Disorder Inventory for Children; BSQ, Body Shape Questionnaire; BDI-II, Beck-Depression-Inventory-II; SCARED, Screen for Child Anxiety Related Emotional Disorders; BID-CA, Test for Body Image Distortion in Children and Adolescents.

### Weight Gain During Inpatient Treatment

The mean values for BMI, BMI-SDS, and BMI age-percentiles for the first 10 weeks of inpatient treatment show the expected weight gain over time (see Table 3). The average increase of BMI over 10 weeks was 2.12 (*SD* = 1.04), of BMI-SDS 1.58 (*SD* = 0.90), and of age-percentiles 6.44 (*SD* = 7.78). After 10 weeks 68.33% had a bodyweight below the 10th age-percentile, while 31.67% had a bodyweight above the 10th age-percentile, 10.00% above the 20th age-percentile, and 5.00% above the 25th age-percentile. Because some patients stayed longer than the survey period, Table 3 additionally presents BMI/BMI-SDS at discharge. At discharge 33.33% had a bodyweight below the 10th age-percentile, while 66.67% had a bodyweight above the 10th age-percentile, 30.00% above the 20th age-percentile, and 25.00% above the 25th age-percentile.

**Table 3:** Descriptive statistics—weight.

**Week**	**BMI**		**BMI-SDS**		**Age-Percentile**	
	***M***	***SD***	***M***	***SD***	***M***	***SD***
0	14.84	1.32	−3.42	1.41	1.33	2.62
1	15.16	1.30	−3.15	1.30	1.77	3.26
2	15.38	1.30	−2.97	1.22	2.09	3.32
3	15.57	1.28	−2.82	1.20	2.50	3.99
4	15.81	1.25	−2.63	1.15	2.99	4.38
5	16.01	1.26	−2.50	1.14	3.59	5.01
6	16.24	1.28	−2.35	1.12	4.38	5.54
7	16.38	1.33	−2.26	1.12	5.05	6.39
8	16.53	1.33	−2.16	1.07	5.61	6.98
9	16.70	1.37	−2.04	1.02	6.42	7.39
10	16.94	1.41	−1.91	0.99	7.88	8.81

*M and SD represent mean and standard deviation, respectively. The age-percentiles smaller than one were coded as 0.1. BMI, Body Mass Index; BMI-SDS, BMI standard deviation score*.

### Influence of Identity Development on Weight Gain

**Figure 1:**
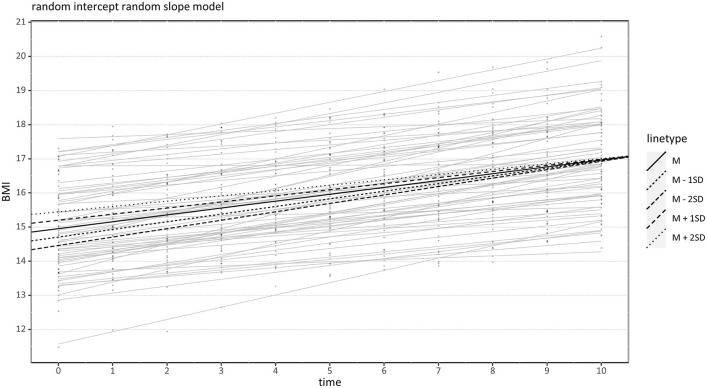
Effect of Assessment of Identity Development in Adolescence (AIDA) on BMI Increase over Time. Black lines indicate conditional regression lines for very low (*M*−2 *SD*), low (*M*−1 *SD*), medium (*M*), high (*M* +1 *SD*), and very high (*M* +2 *SD*) AIDA scores. BMI, Body mass index.

At first, we examined the hypothesis that identity development measured by the AIDA questionnaire negatively influences weight gain (i.e., changes in BMI, BMI-SDS) over time. With respect to the BMI, the ICC was 0.73. The model with random slopes for the time variable had a significantly better model fit than the model with fixed slope, χ^2^(2) = 493.76, *p* < 0.001. That means that individuals with AN substantially differed in their BMI increase within the first 10 weeks. Considering the AIDA as a predictor intercepts and slopes showed a predicted BMI of c_00_ = 14.96 at admission given an average AIDA score, *t*(58) = 89.38, *p* < 0.001, ŝ_0_ = 1.29. Furthermore, given an average AIDA score, the predicted BMI increase per week was c_10_ = 0.20, *t*(58) = 17.45, *p* < 0.001, ŝ_1_ = 0.09. The AIDA score did not significantly influence the predicted BMI at admission, c_01_ = 0.25, *t*(58) = 1.46, *p* = 0.149. However, higher AIDA scores weakened the positive effect of time, c_11_ = −0.02, *t*(58) = −1.96, *p* = 0.027 (one-sided). With other words, individuals with higher identity disturbance gained less weight, as indicated by the BMI, during their first 10 weeks of treatment. In particular, AN patients with a low AIDA score (< M-1SD) gained 2.12 BMI points on average during their first 10 weeks (SD = 1.11), while patients with a high AIDA score (> M+1SD) only gained 1.75 BMI points (SD = 1.12) (see Figure 1). Higher person-specific intercepts were negatively correlated with person-specific slopes (*r* = −0.22) so that patients with higher BMI in the beginning gained less weight over time. Visual inspection of assumptions revealed mostly normally distributed and homoscedastic residuals. A graphical illustration is presented in Figure 1.

**Figure 2:**
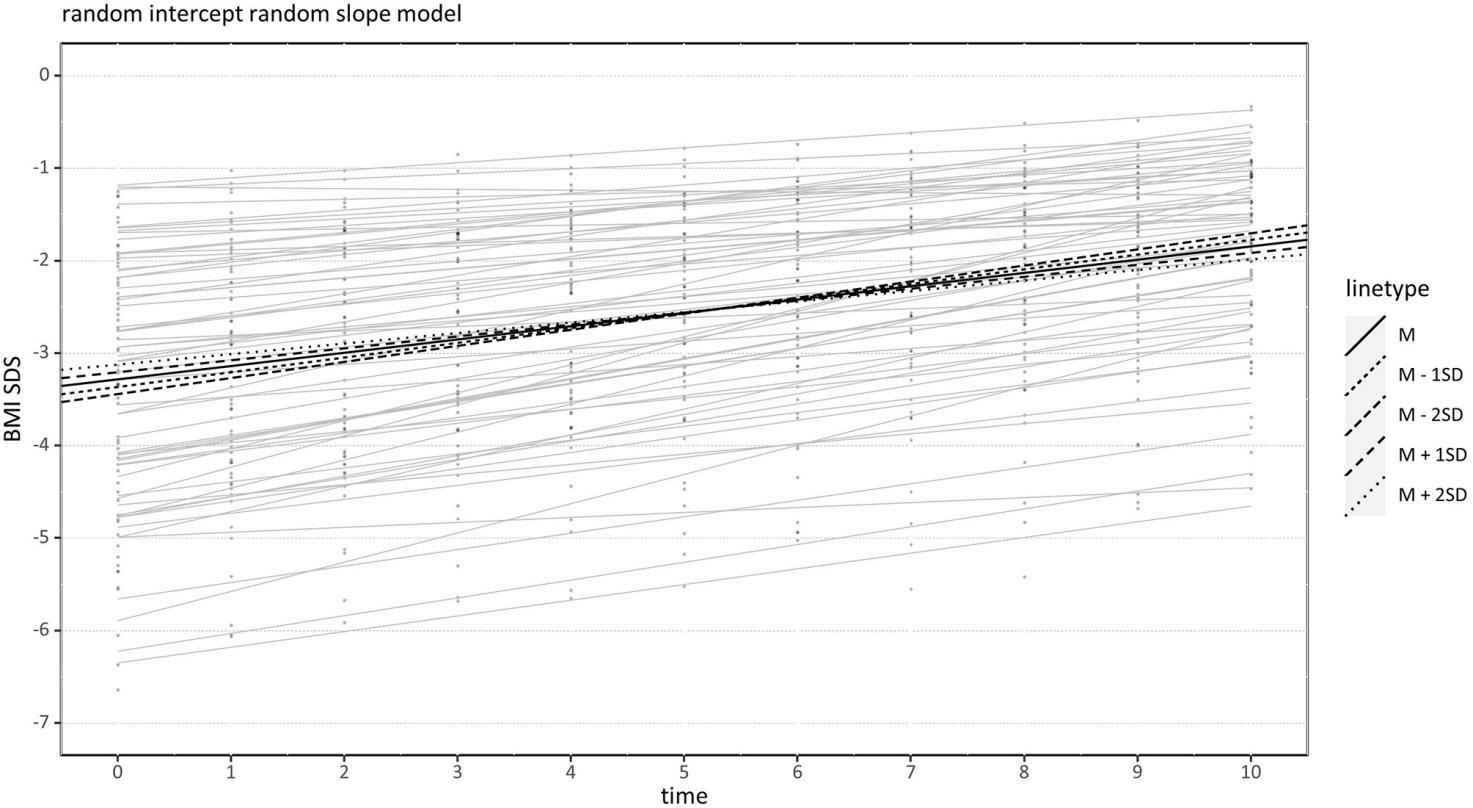
Effect of Assessment of Identity Development in Adolescence (AIDA) on BMI-SDS Increase over Time. Black lines indicate conditional regression lines for very low (*M*−2 *SD*), low (*M*−1 *SD*), medium (*M*), high (*M* +1 *SD*), and very high (*M* +2 *SD*) AIDA scores. BMI-SDS, Body mass index standard deviation score.

With respect to the BMI-SDS, the ICC was 0.80. Again, the random-intercept-random-slope model fitted significantly better than the model with fixed slope, χ^2^(2) = 509.22, *p* < 0.001. When adding the AIDA as a second-level predictor to the model, the predicted BMI-SDS at admission given an average AIDA was c_00_ = −3.28, *t*(58) = −18.74, *p* < 0.001, ŝ_0_ = 1.35. The increase of the predicted BMI-SDS per time point given an average AIDA was c_10_ = 0.14, *t*(58) = 14.69, *p* < 0.001, ŝ_1_ = 0.07. The AIDA score had no significant effect on the predicted BMI-SDS at admission, c_01_ = 0.08, *t*(58) = 0.45, *p* = 0.657. Again, higher AIDA scores weakened the positive effect of time but in contrast to the model with BMI as outcome, the effect was slightly non-significant, c_11_ = −0.015, *t*(58) = −1.51, *p* = 0.068 (one-sided). Again, higher BMI-SDS intercepts were highly negatively correlated with the BMI-SDS increase (*r* = −0.67) so that patients with higher BMI-SDS in the beginning gained less weight over time. So, this effect seems independent of the patient's age. Regarding the assumptions, visual inspection showed slightly non-normal and clearly heteroscedastic residuals. The reason was probably that the BMI-SDS as an outcome variable was left-skewed. Consequently, significance tests should be interpreted with some caution. A graphical illustration is presented in Figure 2.

### Influence of Other Psychopathology Parameters on Weight Gain

In a next step, we examined the hypothesis that identity development is a specifically strong predictor for weight gain (i.e., change of BMI, BMI-SDS) over time. To this end, we repeated the above multilevel analyses using the total scores of all other available psychopathology parameters (for details see Tables 4, 5) to compare them to effects of the AIDA. Not surprisingly, regardless of the significance, the results went into the same direction for all these measures, meaning that higher scores (i.e., more severe psychopathology) were associated with less weight gain. However, the results were unexpected in two ways: First, contrary to our hypothesis, the AIDA had no prominent effect. Several other measures were similar or even stronger negative predictors of weight gain. Interestingly, the two best predictors, found significantly in both models (BMI, BMI-SDS), were measures of body image distortion, i.e., BSQ and BID-CA. Considering that tests were two-sided (vs. one-sided for the AIDA), it might be relevant that also the predictors EDI-C, SCARED and BDI-II (only BMI-SDS) were significant at trend-level. Second, multilevel analyses revealed that several psychopathology parameters were predictive for the BMI and/or BMI-SDS at admission. The two best predictors, found significantly in both models, were again BSQ and BID-CA. Higher initial weight was predicted by higher levels of body image distortion. Moreover, significant predictors of the initial BMI were the EDI-C and BDI-II, and the initial BMI-SDS was predicted by BDI-II at trend level. Again, higher initial weight was associated with higher symptom load.

**Table 4:** Random-intercept-random-slope-model: level-2-predictors and BMI.

**Effect**	**Estimate**	***p***	***t*(df)**	**ŝ**	***r***
**Model 1: EDI-C**
Intercept c_00_	14.76	<0.001	89.01 (58)	1.28	
Time c_10_	0.20	<0.001	17.36 (58)	0.09	−0.27
EDI-C c_01_	0.40	**0.019**	2.42 (58)		
Time*EDI-C c_11_	−0.02	0.080	−1.79 (58)		
**Model 2: BSQ**
Intercept c_00_	14.76	<0.001	98.89 (58)	1.15	
Time c_10_	0.20	<0.001	17.62 (58)	0.09	−0.21
BSQ c_01_	0.69	**<0.001**	4.56 (58)		
Time*BSQ c_11_	−0.03	**0.039**	−2.24 (58)		
**Model 3: BDI-II**
Intercept c_00_	14.76	<0.001	91.91 (58)	1.24	
Time c_10_	0.20	<0.001	17.14 (58)	0.09	−0.28
BDI-II c_01_	0.51	**0.002**	3.17 (58)		
Time*BDI-II c_11_	−0.02	0.207	−1.28 (58)		
**Model 4: SCARED**
Intercept c_00_	14.76	<0.001	85.43 (58)	1.33	
Time c_10_	0.20	<0.001	17.39 (58)	0.09	−0.30
SCARED c_01_	0.16	0.373	0.90 (58)		
Time*SCARED c_11_	−0.21	0.071	−1.84 (58)		
**Model 5: BID-CA**
Intercept c_00_	14.77	<0.001	82.56 (53)	1.32	
Time c_10_	0.20	<0.001	17.24 (53)	0.08	−0.24
BID-CA c_01_	0.40	**0.031**	2.22 (53)		
Time*BID-CA c_11_	−0.03	**0.021**	−2.39 (53)		

*c_00_ describes the predicted BMI/BMI-SDS value at the intercept, c_10_ describes change of the predicted BMI/BMI-SDS value if time is increased by one week, controlled for an average questionnaire score, respectively. Standard deviation s_0_ and s_1_ describe the variation around c_00_ and c_10_, respectively. c_01_ and c_11_ (two-sided in comparison to c_11_ of the AIDA which is one-sided) represent the change in the predicted person-specific intercept and in the predicted change in person-specific slopes, respectively, if the respective psychopathology score is increased by 1 SD. Df represents degrees of freedom. EDI-C, Eating Disorder Inventory for Children; BSQ, Body Shape Questionnaire; BDI-II, Beck-Depression-Inventory-II; SCARED, Screen for Child Anxiety Related Emotional Disorders; BID-CA, Test for Body Image Distortion in Children and Adolescents*.

**Table 5:** Random-intercept-random-slope-model: level-2-predictors and BMI-SDS.

**Effect**	**Estimate**	***p***	***t*(df)**	**ŝ**	***r***
**Model 1: EDI-C**
Intercept c_00_	−3.43	<0.001	−18.99 (58)	1.39	
Time c_10_	0.14	<0.001	14.78 (58)	0.07	−0.69
EDI-C c_01_	0.20	0.285	1.08 (58)		
Time*EDI-C c_11_	−0.02	0.088	−1.74 (58)		
**Model 2: BSQ**
Intercept c_00_	−3.43	<0.001	−19.99 (58)	1.32	
Time c_10_	0.14	<0.001	15.46 (57)	0.07	−0.66
BSQ c_01_	0.48	**0.008**	2.75 (58)		
Time*BSQ c_11_	−0.03	**0.004**	−2.96 (58)		
**Model 3: BDI-II**
Intercept c_00_	−3.43	<0.001	−19.30 (58)	1.37	
Time c_10_	0.14	<0.001	14.74 (58)	0.07	−0.68
BDI-II c_01_	0.32	0.084	1.76 (58)		
Time*BDI-II c_11_	−0.02	0.107	−1.64 (58)		
**Model 4: SCARED**
Intercept c_00_	−3.43	<0.001	−18.86 (58)	1.40	
Time c_10_	0.14	<0.001	14.76 (58)	0.07	−0.70
SCARED c_01_	0.11	0.543	0.61 (58)		
Time*SCARED c_11_	−0.02	0.096	−1.69 (58)		
**Model 5: BID-CA**
Intercept c_00_	−3.46	<0.001	−18.46 (53)	1.39	
Time c_10_	0.14	<0.001	14.75 (53)	0.07	−0.66
BID-CA c_01_	0.40	**0.039**	2.12 (53)		
Time*BID-CA c_11_	−0.03	**0.006**	−2.87 (53)		

*c_00_ describes the predicted BMI/BMI-SDS value at the intercept, c_10_ describes change of the predicted BMI/BMI-SDS value if time is increased by one week, controlled for an average questionnaire score, respectively. Standard deviation s_0_ and s_1_ describe the variation around c_00_ and c_10_, respectively. c_01_ and c_11_ (two-sided in comparison to c_11_ of the AIDA which is one-sided) represent the change in the predicted person-specific intercept and in the predicted change in person-specific slopes, respectively, if the respective psychopathology score is increased by 1 SD. Df represents degrees of freedom. EDI-C, Eating Disorder Inventory for Children; BSQ, Body Shape Questionnaire; BDI-II, Beck-Depression-Inventory-II; SCARED, Screen for Child Anxiety Related Emotional Disorders; BID-CA, Test for Body Image Distortion in Children and Adolescents*.

## Discussion

The present study investigated the influence of identity development on weight gain during inpatient treatment in adolescent patients with AN. To this end, the course of weight gain within the first 10 weeks of treatment (BMI, BMI-SDS) was modeled using multilinear statistics. The analyses used the self-reported level of identity development, indexed by the total score of the AIDA (“diffusion” scale), as a predictor. Moreover, additional analyses explored the predictive value of other indices of illness severity, including ED and comorbid psychopathology. As expected, analyses showed that disturbance of identity development (higher total AIDA scores) negatively influenced individual weight gain. This effect was, however, not particularly strong compared to other indices of illness severity. The strongest predictive effects were found for measures of body image distortion (BID).

### Influence of Identity on Weight Gain

In line with our hypothesis, adolescent AN patient who reported more difficulties accomplishing the age-related task of identity formation showed a less steep course of weight gain. Weight gain is a major goal of AN inpatient treatment (33, 34), and at the end of the study period most patients had not yet achieved their target weight. Thus, slower weight gain within the first 10 weeks of treatment constitutes a clinically meaningful deterioration of the outcome achieved so far. However, in view of its clinical relevance, this effect was moderate (see Figure 1). Moreover, results were significant for BMI as an outcome measure, but only trend-level significant for BMI-SDS. This might be due to a greater statistical uncertainty of the BMI-SDS, particularly regarding extreme values (i.e., severe underweight). Indeed, residuals were not normally-distributed and the *p*-value might be less accurate than in the model with BMI as a dependent variable. Still, the significant predictive effect of AIDA scores on BMI changes confirms that identity disturbance hampered weight gain in adolescent patients with AN. This suggests that difficulties in identity development may impede inpatient treatment success and constitute a relevant aspect of illness severity in AN.

### Influence of Other Indices of Illness Severity on Weight Gain

Explorative analyses of other indices of illness severity as predictors of weight gain revealed that identity disturbance was not a particularly prominent predictor. Indeed, results went in a similar direction, with a negative influence on weight gain, for other psychopathology measures as well (Tables 4, 5). This is in line with prior studies showing that ED and comorbid symptoms were negatively related to weight gain (37, 58). Similar effects of the AIDA and other indices of illness severity might at least in part be explained by relatively high intercorrelations (Table 2). One could assume that identity development and psychopathological symptom levels represent different, but closely related aspects of illness severity in AN.

Interestingly, the two strongest negative predictors of weight gain were two different measures of BID. The BID-CA measures behavioral body size overestimation and covers perceptive aspects of BID (52), while BSQ measures self-reported body dissatisfaction and covers cognitive-affective aspects of BID (46). Further support for the relevance of cognitive-affective BID comes from *post hoc* analyses showing similar predictive effects for the EDI-C subscales “drive for thinness” and “body dissatisfaction” (Supplementary Data 2). A negative influence of BID on the course of AN, especially regarding weight gain, was found by several other studies as well [perceptive: (38, 59)] [cognitive-affective: (35, 59, 60)]. These convergent results including both perceptive and cognitive-affective aspects of BID strengthen the assumption that BID is highly relevant to weight gain during inpatient treatment. Interestingly, AIDA and BID-CA were uncorrelated, suggesting that identity disturbance and perceptive BID^1^ are at least partially independent, complementary aspects that are relevant for the prediction of treatment success.

Of course, these explorative analyses have to be interpreted with caution. However, we think that the results raise an interesting issue: It might be speculated that identity disturbance and other forms of psychopathology influence each other, i.e., coexisting psychopathology (including symptoms of AN) might interfere with identity development and/or identity disturbance might exacerbate psychopathology. One could assume that these predictors are difficult to separate, and collectively contribute to a worse treatment outcome.

### Relationship With Weight at Admission

A further revealing result is the negative correlation between higher weight at the beginning of treatment and weight gain over time (Figures 1, 2). If AN patients suffered from less severe starvation at the beginning of treatment, their weight gain was smaller. This was not because there was no need for further weight gain: After 10 weeks of treatment only three AN patients had achieved their target weight, while 57 AN patients still followed the treatment regime including a weekly weight gain of 500 g. Several other studies reported the phenomenon of initial low weight resulting in rapid weight gain and initial high weight resulting in slower weight gain as well (37, 58, 62). This observation contrasts with the DSM-5 definition of the degree of underweight as a central indicator of illness severity in AN, which might lead to the expectation that more severe underweight is associated with a poorer course of treatment and less weight gain. Several reasons for the observed opposite effect are discussed: Individuals with lower weight might gain more weight when eating the same amount of calories (37, 63). Moreover, the staff could be more attentive to individuals with lower weight (37), and, specifically in adolescent patients who are often more extrinsically motivated, a higher weight at admission was linked to less motivation to gain weight (58). Thus, AN patients with a higher weight at admission may experience less pressure (e.g., from parents, staff, or based on own concerns) and thus be less motivated to gain weight. Whatever the reason, these results are in line with the clinical experience that the adolescent AN patients with initially severe underweight are not necessarily the same patients who are struggling with weight gain. It might be suggested to subdivide the term “illness severity” into the aspects “current somatic threat” and “psychopathology”, with the latter probably having more relevance to prognosis.

Besides this general relationship of initial weight and weight gain, a higher weight at admission was also predicted by several psychopathology measures. Both BMI and BMI-SDS at admission were predicted by the two measures of BID (BSQ, BID-CA). Moreover, BMI at admission was predicted by ED symptoms and depressive symptoms (EDI-C, BDI-II). This means that, convergent with prior findings (64), AN patients with a higher initial weight reported more severe psychopathology, in particular BID. One possible reason is that individuals with higher initial weight are further away from their ideal body image (65). Alternatively, the patients with severe underweight might suffer more from alexithymia, i.e., difficulty in identifying and describing feelings. Alexithymia was observed in almost three-quarters of the individuals with AN (66, 67) and it decreases during weight restoration (68–70). Thus, AN patients with more severe underweight might show higher levels of alexithymia, which leads to reduced self-reports of psychopathology. On the other hand, ED patients with high levels of alexithymia were reported to show higher levels of specific ED psychopathology and, despite noticeable improvements, to remain at clinically relevant alexithymia scores at the end of inpatient treatment (70). Thus, alexithymia could also be a rather stable personality trait. More data would be needed to clarify a possible relationship between alexithymia, ED psychopathology and underweight. Further research on this topic might be worthwhile, e.g., parallel assessment of alexithymia and psychopathology at multiple time points during weight gain.

One more aspect is relevant to the interpretation of the present data: The prediction of both the initial weight and the course of weight gain by the same psychopathology measures. AN patients who reported higher levels of psychopathology (specifically BID) had both higher initial BMI/BMI-SDS and slower weight gain. As patients with higher BMI/BMI-SDS at admission had slower weight gain in general, the weakening effect of BID (and other psychopathology measures) on weight gain might be at least partly explained by the higher initial weight. This would mean that higher levels of psychopathology *per se* have no or only a small direct effect on weight gain. Importantly, the AIDA, which was the main focus of our study, did not predict weight at admission. This suggests that the influence of higher AIDA scores on weight gain cannot be explained by this alternative pathway and points to a certain degree of specificity of identity disturbance as a predictor of inpatient treatment success.

### Limitations and Further Research

The study has several limitations. First, the study includes a moderate sample size of only female individuals, which limits generalizability of the results. Second, we excluded individuals who were discharged before reaching 10 weeks of hospitalization. As these are probably individuals with extremely positive or negative courses, this could bias the results. Third, identity disturbance and other psychopathology were measured by self-report questionnaires. Future studies may supplement this approach with assessments from therapists, e.g., outcome ratings and clinical interviews. Related to this, all self-report questionnaires were filled in within the first treatment phase, but after an average of about 2 weeks. Unfortunately, the existing clinical routines did not allow for a more near-term measurement. During this time, some treatment effects could already have occurred, and this might have biased our data. However, given that the treatment of AN typically takes considerably longer, we do not expect major treatment-related changes. Fourth, we used weight gain during the first 10 weeks as a progression parameter. To confirm and expand our results, more parameters should be analyzed during the course of treatment, such as psychopathology and alexithymia. Furthermore, the influence of identity on other parameters apart from weight gain, such as drop-out rate, long-term treatment compliance, and relapse should be examined.

## Conclusion

The present study revealed that, during inpatient treatment of adolescents with AN, weight gain was negatively predicted by the level of identity functioning, indicated by the AIDA. Thus, adolescent AN patients who reported to have difficulty developing a stable identity struggled more with gaining weight. Interestingly, severe underweight at admission was associated with better weight gain. Thus, the level of underweight at admission might have greater importance for acute aspects of illness severity, like somatic threat. Inversely, initially higher weight was associated with both worse weight gain and higher levels of psychopathology. Thus, the fact that psychopathology, and especially BID, predicted less weight gain, might at least partly be explained by a higher initial weight in those with higher symptom levels. Based on this data, it is difficult to say what should be considered more important for a clinical prognosis of weight gain: initial weight (with higher weight predicting worse outcome) or psychopathology (with higher symptom levels predicting worse outcome). This is different for the level of identity functioning, as it predicted weight gain independently of the initial weight. Thus, difficulties in identity development may impede inpatient treatment success independently of the initial weight and seem to constitute a relevant aspect of illness severity in AN. This recommends a greater consideration of identity development during treatment.

## Data Availability Statement

The raw data supporting the conclusions of this article will be made available by the authors, without undue reservation.

## Ethics Statement

The studies involving human participants were reviewed and approved by Ethics Committee of the Medical Association of Westphalian-Lippe and the University of Münster. Written informed consent from the participants' legal guardian/next of kin was not required to participate in this study in accordance with the national legislation and the institutional requirements.

## Author Contributions

LB and IW: conception and design. LB: data preparation and analysis, and wrote first draft of the article. SW: statistical analysis including the writing part and critical review of the text with a focus on methodological aspects. MF: critical review of the text with a focus on clinical aspects. AD: data collection of the BID-CA and critical review of the text with a focus on clinical aspects. JM: critical review of the text with a focus on methodological aspects. IW: critical review of the text. All authors contributed to the article and approved the submitted version.

## Funding

Parts of the data included in this study were collected as part of a project funded by the German Research Foundation (Project Number: WE 6188/2-1).

## Conflict of Interest

The authors declare that the research was conducted in the absence of any commercial or financial relationships that could be construed as a potential conflict of interest.

## Publisher's Note

All claims expressed in this article are solely those of the authors and do not necessarily represent those of their affiliated organizations, or those of the publisher, the editors and the reviewers. Any product that may be evaluated in this article, or claim that may be made by its manufacturer, is not guaranteed or endorsed by the publisher.

## Footnotes

^1^Recent literature confirms AN patients' overestimation in body size estimation tasks but questions the *perceptive* nature of this phenomenon (61). Alternatively, compared to aspects of BID captured by questionnaires, i.e. explicit judgements, the BID-CA might capture more implicit aspects of BID. Relatively stronger correlations between AIDA and BSQ and lower correlations between AIDA and BID-CA might thus reflect differences between explicit (questionnaire) versus more implicit (behavioral) aspects and/or measurement methods.
