# Supplementary material for: Influence of Identity Development on Weight Gain in Adolescent Anorexia Nervosa
Source: Front Psychiatry. 2022 May 26;13:887588. doi: 10.3389/fpsyt.2022.887588 (PMC9186337; doi:10.3389/fpsyt.2022.887588)
Supplement: Supplementary file 1 [file Data_Sheet_1.docx]

Supplementary Material

**Supplementary Table 1** Comorbidities

| Diagnosis | Frequency |
| --- | --- |
| major depressive disorder (MDD) | n = 24 |
| anxiety disorder | n = 9 |
| obsessive-compulsive disorder | n = 3 |
| posttraumatic stress disorder | n = 1 |
| gender dysphoria | n = 1 |
| avoidant personality disorder | n = 1 |
| specific learning disorder | n = 2 |

*Note*. Diagnosis according to DSM-5. The category anxiety disorder consists of social anxiety disorder, generalized anxiety disorder, specific phobia, and panic disorder.

**Supplementary Data 1** Clinical Classification of Questionnaires and Body Size Estimation Task

Mean values for the different questionnaires were calculated, see Table 2. To analyze the results, it is important to classify them according to their clinical meaning.

The AIDA questionnaire enables to classify between healthy identity development (T < 60), identity crisis (T > 60) and identity diffusion (T > 70) (9). In our sample of AN patients, the mean T-value was 59.62 (*SD* = 11.39) for the total scale “diffusion” and the mean T-values for the two main subscales were 61.60 (*SD* = 11.97) for “discontinuity”, and 56.88 (*SD* = 10.81) for “incoherence”. Considering the observed standard deviations, our sample included patients showing healthy identity development, as well as acute crisis and diffusion.

The EDI-C does not allow a normative evaluation of the total score used in our analyses. However, standardized percentile ranks are available for the subscales (45), and to improve comparability these were additionally converted to T-values^[[1]](#footnote-1)^. For a general overview on clinical relevance, the mean values of each EDI-C subscale were converted to normative values for 15-year-old girls (sample age *M* = 15.35, *SD* = 1.78):

Drive for thinness M = 22,28, SD = 10.27, PR = 85, T = 60.4

Bulimia M = 6.88, SD = 3.65, PR = 75, T = 56.7

Body dissatisfaction M = 29.90 SD = 10.04, PR = 75, T = 56.7

Ineffectiveness M = 27.90, SD = 9.54, PR = 85, T = 60.4

Perfectionism M = 13.27, SD = 5.54, PR = 80, T = 58.4

Interpersonal distrust M = 14.55, SD = 6.16, PR = 75, T = 56.7

Interoceptive awareness M = 20.33, SD = 8.40, PR = 75, T = 56.7

Maturity fears M = 20.07, SD = 6.32, PR = 70, T = 55.2

Asceticism M = 22.85, SD = 6.03, PR = 85, T = 60.4

Impulse regulation M = 18.27, SD = 6.40, PR = 65, T = 53.9

Social insecurity M = 19.15, SD = 6.94, PR = 80, T = 58.4

The BSQ is also standardized in percentile ranks (47), which were additionally converted to T-values here2. The mean percentile in this study was 82.27 (*SD* = 26.08), corresponding to a T-value of 63.76 (*SD* = 11.82). This indicates an overall enhanced body dissatisfaction.

The clinical evaluation of the BDI-II is achieved using limit values that indicate no, minimal, moderate or severe depression. The mean BDI-II value of our sample (M = 24.48, SD = 10.74) can be classified as a moderate depression (range 20-28) (49). In view of the observed standard deviation it can be assumed that our sample included AN patients without clinically relevant depression, as well as with minimal, moderate and severe levels of depression. Accordingly, major depression was the most frequent comorbid diagnosis.

In the SCARED questionnaire a total score of >25 indicates a potential anxiety disorder. This limit is exceeded by the mean value of 29.52 (*SD* = 13.64) observed in this study (50,51). These elevated values were only partly reflected by comorbid anxiety disorder diagnoses (n = 9, 15.00%). However, elevated anxiety levels might also be related to AN (e.g. fear of gaining weight) and other comorbid disorders (OCD, PTSD, avoidant personality disorder).

For the BID-CA there are no normative values available. Based on a sample of healthy female adolescents (mean age of 15.1, *SD* = 2.1) one can assume a normal range (mean ± 1 *SD*) for body image distortion between 95.2 and 139.2 for BID-I_arm_, between 99.8 and 135.2 for BID-I_waist_, and between 92.9 and 124.9 for BID-I_thigh_ (52). In this study the mean BID-I_arm_ was 124.93 (*SD* = 17.98) and can thus be considered as lying within the normal range. The mean BID-I_waist_ was 137.50 (*SD* = 20.93), and the mean BID-I_thigh_ 131.96 (*SD* = 21.75). Both values exceeded the normal range, indicating that – on average – the AN patients in this study showed a significant body size overestimation of the waist and thigh.

**Supplementary Data 2** Multilevel Analyses - Subscales EDI-C

**Table S1** Random-Intercept-Random-Slope-Model: Level-2-Predictors “drive for thinness” (DT) and “body dissatisfaction” (BD) and BMI

| Model S1: EDI-C drive for thinness (DT) | | | | | |
| --- | --- | --- | --- | --- | --- |
| Effect | Estimate | *p* | *t*(df) | $\hat{s}$ | *r* |
| Intercept c_00_ | 14.96 | < .001 | 93.18 (58) | 1.24 |  |
| Time c_10_ | 0.20 | < .001 | 17.33 (58) | 0.09 | -.19 |
| DT c_01_ | 0.44 | **.009** | 2.71 (58) |  |  |
| Time*DT c_11_ | -0.02 | .089 | -1.73 (58) |  |  |
| Model S2: EDI-C body dissatisfaction (BD) | | | | | |
| Effect | Estimate | *p* | *t*(df) | $\hat{s}$ | *r* |
| Intercept c_00_ | 14.96 | < .001 | 95.86 (58) | 1.20 |  |
| Time c_10_ | 0.20 | < .001 | 17.62 (58) | 0.09 | -.16 |
| BD c_01_ | 0.53 | **.001** | 3.42 (58) |  |  |
| Time*BD c_11_ | -0.03 | **.028** | -2.26 (58) |  |  |

*Note.* c_00_ describes the predicted BMI/BMI-SDS value at the intercept, c_10_ describes change of the predicted BMI/BMI-SDS value if time is increased by one week, controlled for an average questionnaire score, respectively. Standard deviation s_0_ and s_1_ describe the variation around c_00_ and c_10_, respectively. c_01_ and c_11_ (two-sided) represent the change in the predicted person-specific intercept and in the predicted change in person-specific slopes, respectively, if the respective psychopathology score is increased by 1 *SD*. *Df* represents degrees of freedom.

**Table S2** Random-Intercept-Random-Slope-Model: Level-2-Predictors “drive for thinness” (DT) and “body dissatisfaction” (BD) and BMI**-**SDS

| Model S1: EDI-C drive for thinness (DT) | | | | | |
| --- | --- | --- | --- | --- | --- |
| Effect | Estimate | *p* | *t*(df) | $\hat{s}$ | *r* |
| Intercept c_00_ | -3.27 | < .001 | -19.00 (58) | 1.33 |  |
| Time c_10_ | 0.14 | < .001 | 14.82 (58) | 0.07 | -.66 |
| DT c_01_ | 0.24 | .173 | 1.38 (58) |  |  |
| Time*DT c_11_ | -0.02 | .070 | -1.85 (58) |  |  |
| Model S2: EDI-C body dissatisfaction (BD) | | | | | |
| Effect | Estimate | *p* | *t*(df) | $\hat{s}$ | *r* |
| Intercept c_00_ | -3.27 | < .001 | -19.22 (58) | 1.32 |  |
| Time c_10_ | 0.14 | < .001 | 15.30 (58) | 0.07 | -.65 |
| BD c_01_ | 0.31 | .074 | 1.82 (58) |  |  |
| Time*BD c_11_ | -0.03 | **.008** | -2.73 (58) |  |  |

*Note.* c_00_ describes the predicted BMI/BMI-SDS value at the intercept, c_10_ describes change of the predicted BMI/BMI-SDS value if time is increased by one week, controlled for an average questionnaire score, respectively. Standard deviation s_0_ and s_1_ describe the variation around c_00_ and c_10_, respectively. c_01_ and c_11_ (two-sided) represent the change in the predicted person-specific intercept and in the predicted change in person-specific slopes, respectively, if the respective psychopathology score is increased by 1 *SD*. *Df* represents degrees of freedom.

1. Calculated at https://www.psychometrica.de/normwertrechner.html [↑](#footnote-ref-1)
